# Identification and characterization of the LDAP family revealed *GhLDAP2_Dt* enhances drought tolerance in cotton

**DOI:** 10.3389/fpls.2023.1167761

**Published:** 2023-05-16

**Authors:** Yanyan Zhao, Bailin Duan, Yuxin Liu, Yuqing Wu, Dongliang Yu, Liping Ke, Fangfang Cai, Jun Mei, Ning Zhu, Yuqiang Sun

**Affiliations:** Plant Genomics and Molecular Improvement of Colored Fiber Lab, College of Life Sciences and Medicine, Zhejiang Sci-Tech University, Hangzhou, China

**Keywords:** LDAP, cotton, drought stress, gene duplication, gene identification

## Abstract

Lipid droplet-associated proteins (LDAPs) play essential roles in tissue growth and development and in drought stress responses in plants. Cotton is an important fiber and cash crop; however, the LDAP family has not been characterized in cotton. In this study, a total of 14, six, seven, and seven genes were confirmed as LDAP family members in *Gossypium hirsutum*, *Gossypium raimondii*, *Gossypium arboreum*, and *Gossypium stocksii*, respectively. Additionally, expansion in the LDAP family occurred with the formation of *Gossypium*, which is mirrored in the number of LDAPs found in five Malvaceae species (*Gossypioides kirkii*, *Bombax ceiba*, *Durio zibethinus*, *Theobroma cacao*, and *Corchorus capsularis*), *Arabidopsis thaliana*, and *Carica papaya*. The phylogenetic tree showed that the *LDAP* genes in cotton can be divided into three groups (I, II, and III). The analysis of gene structure and conserved domains showed that *LDAPs* derived from group I (*LDAP1*/*2*/*3*) are highly conserved during evolution, while members from groups II and III had large variations in both domains and gene structures. The gene expression pattern analysis of *LDAP* genes showed that they are expressed not only in the reproductive organs (ovule) but also in vegetative organs (root, stem, and leaves). The expression level of two genes in group III, *GhLDAP6_At/Dt*, were significantly higher in fiber development than in other tissues, indicating that it may be an important regulator of cotton fiber development. In group III, *GhLDAP2_At*/*Dt*, especially *GhLDAP2_Dt* was strongly induced by various abiotic stresses. Decreasing the expression of *GhLDAP2_Dt* in cotton *via* virus-induced gene silencing increased the drought sensitivity, and the over-expression of *GhLDAP2_Dt* led to increased tolerance to mannitol-simulated osmotic stress at the germination stage. Thus, we conclude that *GhLDAP2_Dt* plays a positive role in drought tolerance.

## Introduction

Upland cotton (*Gossypium hirsutum* L.) is widely cultivated as an important fiber crop globally (Ke et al., 2022; Mei et al., 2022). Cotton fibers can be woven, knitted, or felted to create a wide variety of fabrics. Xinjiang has a typical continental arid climate, with abundant sunshine and abundant heat (Yu et al., 2021). These special climatic conditions provide unique natural conditions for cotton planting, and Xinjiang has become the largest high-quality cotton production base in China. However, as a typical arid region in the north temperate zone, the ongoing water scarcity has severely impacted its cotton production. Thus, it is of utmost importance to uncover the molecular mechanisms of cotton in response to drought stress and breed drought-tolerant cotton cultivars.

Cytoplasmic lipid droplets (LDs) act as storage organelles with signaling roles during development and stress (Müller et al., 2017; Huang, 2018; Kretzschmar et al., 2020; Huang et al., 2022). These functions can be accomplished by storing functional molecules such as triacylglycerols and sterol esters (Richardson, 2020). Structurally, LDs consist of a neutral lipid core that is uniquely wrapped by a single phospholipid monolayer and coated with a variety of “coat” proteins that either bind directly to the LD surface from the cytoplasm or target the LD surface *via the* endoplasmic reticulum (ER) (Gidda et al., 2016). Among these LD-associated proteins, oleosins were the first family of LD proteins discovered in plant and have been well characterized (Shao et al., 2019). Oleosins can promote the formation of LDs *via* budding from the ER’s outer leaflet, as well as preventing the fusion of mature LDs during seed desiccation (Huang and Huang, 2015). Oleosins, however, are predominantly expressed in seeds and pollen grains, and they are almost absent in the vegetative tissues (Shimada et al., 2008; Lévesque-Lemay et al., 2016). There is emerging evidence that LDs also play important roles in physiological processes within vegetative tissues; thus, it is essential to gain insight into LD-associated proteins in vegetative tissues (Horn et al., 2013; Pyc et al., 2017).

The discovery of the lipid droplet-associated protein (LDAP) family has gained insight into the biogenesis and function of LDs in non-seeded tissues, which are characterized by a conserved REF domain (Horn et al., 2013; Brocard et al., 2017). Rubber elongation factor (REF) proteins were first identified in rubber particles, which contain small rubber particle protein (SRPP) and a smaller REF homolog, both of which promote rubber biosynthesis (Tang et al., 2016; Tong et al., 2017). LDAPs share a high sequence similarity with SRPP/REF proteins in rubber-accumulating plants, suggesting that LDs are rubber particle-like organelles that compartmentalize TAGs rather than polyisoprenes in non-rubber-producing plants (Pyc et al., 2021; Nam et al., 2022). Because of the close involvement of REF/SRPP proteins in rubber synthesis, their functions in rubber-producing plants are more intensively studied. In non-rubber-producing plants, these SRPP-like proteins were referred to as LDAPs (Gidda et al., 2016; Kretzschmar et al., 2020; Richardson, 2020). LDAP protein was originally identified in the mesocarp tissue of avocado (Horn et al., 2013). Subsequently, three ubiquitously expressed LDAP family members, *LDAP1*–*3*, were identified in *Arabidopsis* (Kim et al., 2016).

There is growing evidence that LDs play important roles in both the biotic and abiotic stress responses of plants (Kim et al., 2016; Yang and Benning, 2018). LD proliferation is a common cellular response in response to different abiotic stresses, and *LDAPs* are critical for LD proliferation during stress-related processes—for example, in *Arabidopsis*, the loss of *LDAP3* resulted in fewer LDs in response to cold in comparison to the wild type, and the reduction of *LDAP1* expression resulted in the reduced proliferation of LDs under heat stress (Kim et al., 2016; Pyc et al., 2021). Prior studies revealed that the *LDAP* genes and their SRPP homologs are strongly induced by abiotic stresses—for instance, among the five *SRPP* genes in *Taraxacum brevicorniculatum*, all except *TbSRPP4* and *TbSRPP5* were upregulated in response to abiotic stress, and transgenic *Arabidopsis* that overexpressed *TbSRPP2* and *TbSRPP3* exhibited better drought stress tolerance than wild-type plants (Laibach et al., 2018). In *Capsicum annuum*, the overexpression of *CaLDAP1* resulted in enhanced tolerance to drought stress in comparison to the control plants (Kim et al., 2010).

Although the function of *LDAPs* has been reported in some species, little attention has been paid to the LDAP family in cotton. In the current study, we identified the *LDAPs* in four cotton species, including drought-tolerant diploid wild species (*G. stocksii*), allotetraploid cotton (*G. hirsutum*), and its diploid progenitors (*G. arboreum* and *G. raimondii*). Their features, including evolution, gene structure, expression patterns, and biological function, were further analyzed. We then investigated the role of *GhLDAP2_Dt in* drought stress. Overall, these results provide an understanding of the function of *LDAPs* in cotton.

## Materials and methods

### Identification of cotton *LDAP* genes

The genome sequences of the species used in this study were downloaded from public databases. The specific data sources for these genomic sequences are listed in **Supplementary Table S1**. Firstly, we used the LDAP protein sequences from *A. thaliana* as queries to search in the genome database with BLASTP search. Secondly, proteins containing the REF domain (PF05755) in the genome database were identified using the hidden Markov model search. Finally, all the non-redundant LDAP protein sequences were further identified using the following tools: Pfam (http://pfam.xfam.org/), SMART (http://smart.embl.de/smart/set_mode.cgi?NORMAL=1), and Batch CD-Search (https://www.ncbi.nlm.nih.gov/Structure/cdd/cdd.shtml).

### Phylogenetic tree and gene structure analysis

For the phylogenetic analysis, the full-length LDAP protein sequences were aligned using ClustalW program (build-in MEGA 11) (Tamura et al., 2021), and a neighbor-joining tree was built using the bootstrap method with 1,000 replicates. The exon position was acquired from the gff3 file. The MEME Suite (http://meme-suite.org/tools/meme) was used to analyze the conserved motifs of cotton LDAP proteins using the following parameters: motif width that ranged from 6 to 200 residues and the maximum number of motifs equal to 10. Finally, the gene structure and conserved domains of LDAPs were displayed using TBtools (Chen et al., 2020).

### Synteny and gene duplications analysis

Firstly, the whole genome protein sequences of *G. kirkii*, *T. cacao*, and the three cotton species (*G. raimondii*, *G. arboreum*, and *G. hirsutum*) were pairwise compared using BLAST. Then, a synteny analysis of inter- and intra-species *LDAP* genes was performed using MCScanX (Wang et al., 2012). We also used the MCScanX software to estimate gene duplication event types in these species.

### Plant materials

*G. hirsutum* L. cv. Coker 312 (C312) was planted in a field in Hangzhou, Zhejiang. At the three-leaf stage, the seedlings were exposed to polyethylene glycol (PEG) 6000 solution between 0 and 24 h. The leaves were harvested at 0, 1, 3, 6, and 12 h post-treatment. The samples were then frozen in liquid nitrogen and stored at -80°C for RNA extraction and subsequent analysis. The *GhLDAP2_Dt* coding region was amplified using specific primers p2300-GhLDAP2F and p2300-GhLDAP2R (see **Supplementary Table S1**) and cloned into the XbaI and KpnI sites of the pCambia2300 vector to generate the 35S::GhLDAP2_Dt construct. The construct was transformed into *Agrobacterium tumefaciens* strain GV3101 and then transformed into wild-type *Arabidopsis thaliana* Columbia ecotype (Col-0) plants by the floral dip method (Zhao et al., 2019).

### Virus-induced gene silencing of *GhLDAP2_Dt*

The cotton leaf crumple virus (CLCrV)-based virus-induced gene silencing (VIGS) vectors were used to silence *GhLDAP2_Dt in* cotton (Gu et al., 2014). A specific fragment of GhLDAP2_Dt was cloned into the SpeI and AscI restriction sites of the CLCrV-based vector to generate pCLCrV-*GhLDAP2_Dt*, and pCLCrV-*CHLI* (cotton magnesium chelatase subunit I) was used as a positive control. The specific fragment was amplified from C312 cDNA using the primers CLCRV-LDAP2-F/CLCRV-LDAP2-R. The vectors were subsequently transformed into *Agrobacterium tumefaciens* strain GV3101. The cotyledons of 7-day-old C312 cotton seedlings were then injected with equal amounts of *Agrobacterium* expressing the CLCrV vectors as previously described. Real-time PCR was used to check the interference efficiency using primers GhLDAP2-F and GhLDAP2-R (**Supplementary Table S2**). Cotton *Ubiquitin7* gene (*GhUBQ7*, accession number: DQ116441) was used as an internal control.

### Stress tolerance assays

For the germination assay, sterilized seeds of wild-type and *GhLDAP2_Dt* transgenic *Arabidopsis* were planted on MS medium saturated with 400 mM mannitol and incubated at 4°C for 48 h before being transferred to 22°C under a 16/8-h light/dark regime. The seeds were considered germinated when radicles completely penetrated the seed coat. Germination was scored daily up to 10 days to calculate the germination rate. Growth was monitored using cotyledon greening rate on the 10th day post-germination. For the drought tolerance assay, control and *GhLDAP2_Dt*-silenced plants were subjected to natural drought treatment when the plants produced the third true leaf for approximately 3 weeks, after which watering was resumed.

### Gene expression pattern analysis

The expression levels (fragments per kilobase per million reads, FPKMs) of the LDAP family were extracted from the high-throughput *G. hirsutum* TM-1 transcriptome sequencing data (PRJNA490626) (Zhang et al., 2015). The raw RNA-seq data of *G. stocksii* and *G. arboreum* were downloaded from NCBI under project PRJNA712942 (Yu et al., 2021). Heat map charts were generated based on the FPKM values, and images were created using TBtools software (Chen et al., 2020). Quantitative real-time polymerase chain reaction (qRT-PCR) was performed as previously described (Zheng et al., 2021). The gene-specific primers used in the qRT-PCR are listed in **Supplementary Table S2**. The 2^−ΔΔCt^ method was applied to calculate the relative gene expression levels.

## Results

### Identification of *LDAP* genes in four cotton species

**Figure 1:**
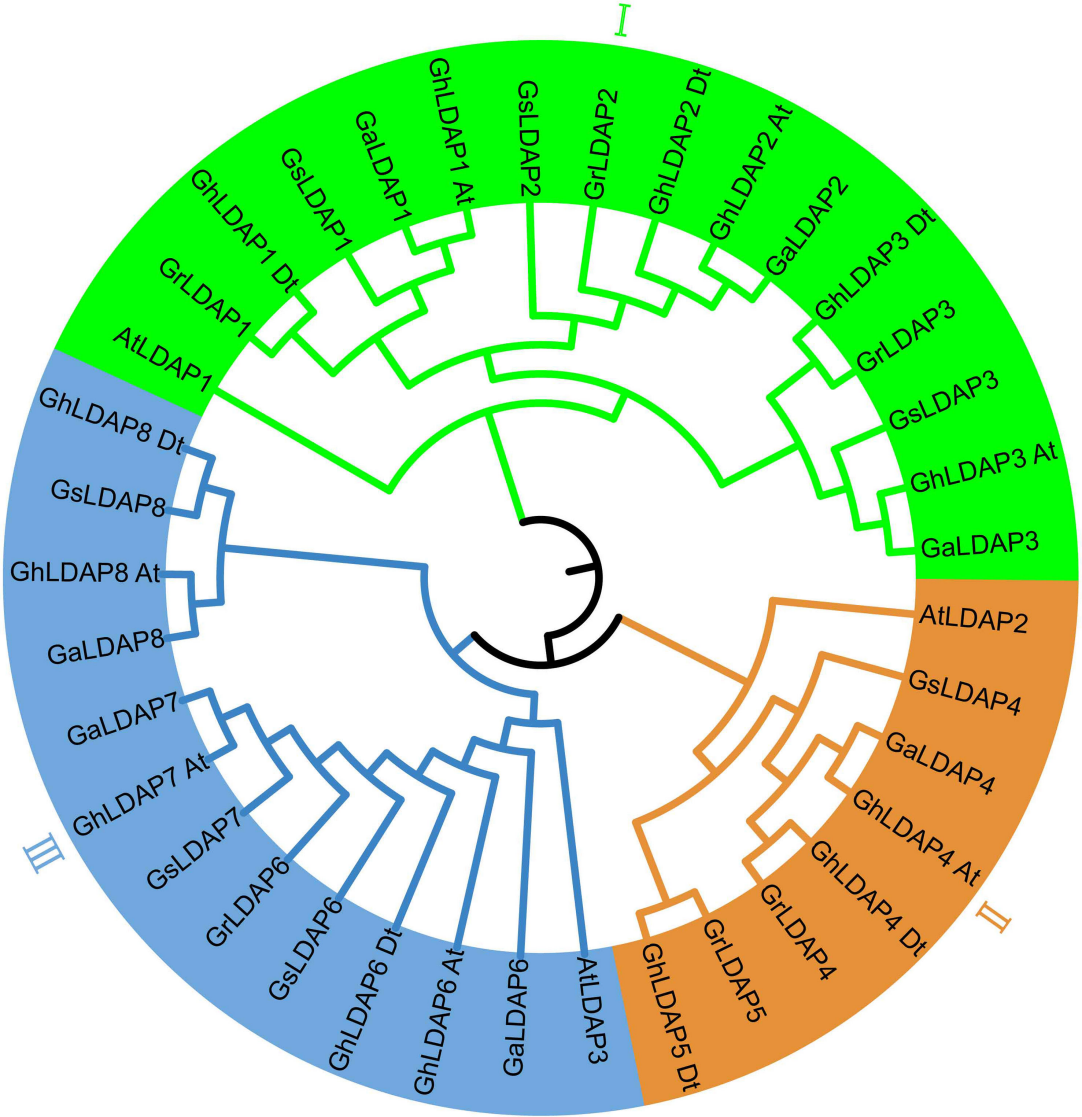
Phylogenetic tree of lipid droplet-associated proteins in *Arabidopsis* and species of cotton. The outer circle is marked in green, brown, and blue, which represent groups I, II, and III, respectively.

In the combined results of BLASTP search, hmmsearch, and CDD check, a total of 14, six, seven, and seven genes were confirmed as LDAP family members in *G. hirsutum*, *G. raimondii*, *G. arboreum*, and *G. stocksii*, respectively. The gene ID and physical location of these *LDAPs* are listed in **Supplementary Table S3**. To study the phylogenetic relationship of *LDAP* genes among *A. thaliana and* cotton species, a phylogenetic tree was constructed using MEGA 11. These genes were grouped and named according to their phylogenetic relationships. The phylogenetic tree showed that the *LDAP* genes can be divided into three groups (I, II, and III) (**Figure 1**). Each group contains one *Arabidopsis LDAP* gene and multiple cotton *LDAP* genes. In group I, the genes in three diploid cottons (*G. raimondii*, *G. arboreum*, and *G. stocksii*) showed a one-to-one correspondence. However, in group III, *GrLDAP7 and GrLDAP8* were absent from the clustering of their corresponding homologous genes in *G. arboreum* and *G. stocksii*. In group II, *LDAP5* only existed in the D genome (*G. raimondii*) and Dt-subgenome (*G. hirsutum*), which might have arose from independent gene expansion events in D/Dt-subgenome.

### LDAP family expansion with the *Gossypium* formation

**Figure 2:**
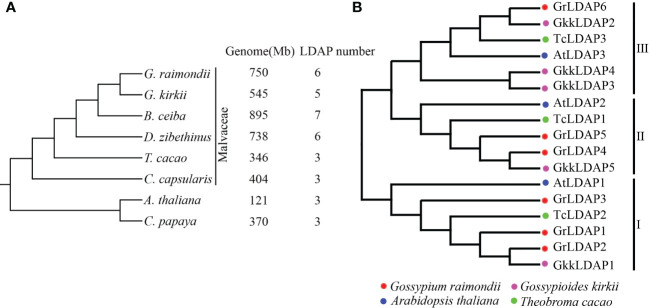
*LDAP* gene family expansion with the *Gossypium* formation. **(A)** The tree topology reflects the inferred phylogenetic analysis among Malvaceae species (*G. kirkii*, *B.ceiba*, *D.zibethinus*, *T. cacao*, and *C. capsularis*), *A. thaliana*, and *C. papaya*. The genome size and LDAP gene numbers for each species are shown separately on the right. **(B)** Phylogenetic analysis of lipid droplet-associated proteins in *A. thaliana*, *G. raimondii*, *T. cacao*, and *G. kirkii.*.

Since the copy number of genes may play a dose–effect when functioning, we investigated whether the amplification of *LDAP* genes accompanied the evolution of cotton. To elucidate the evolutionary history of the LDAP family, we observed the *LDAP* gene numbers in other complete-sequenced Malvaceae species (*G. kirkii*, *B. ceiba*, *D. zibethinus*, *T. cacao*, and *C. capsularis*), *A. thaliana*, and *C. papaya*. The gene ID and physical location of these *LDAPs* are listed in **Supplementary Table S4**. Their phylogeny trees were performed based on single-copy orthologous genes (**Figure 2A**). The results show that the LDAP family underwent expansion in *G. raimondii*, *G. Kirkii*, *B. ceiba*, and *D. zibethinus* compared with those in other species (*T. cacao*, *A. thaliana*, *C. capsularis*, and *C. papaya*), which is mirrored in the number of *LDAPs* found in these species (**Figure 2A**). *G. kirkii* and *G. raimondii* are the closest relatives among these species (**Figure 2A**). To better understand the process of LDAP family expansion accompanied with *G. kirkii* or *G. raimondii* formation, the LDAP proteins identified from *G. raimondii*, *G. Kirkii*, *T. cacao*, and *A. thaliana* were aligned to construct a phylogenetic tree. As shown in **Figure 2B**, these LDAP proteins were divided into three distinct groups (I, II, and III). Each group contains one LDAP from *A. thaliana* and one LDAP from *T. cacao*, whereas in groups I and II, one LDAP from *A. thaliana* corresponds to more than one homologous gene from *G. raimondii*, and in group III, the number of LDAP genes in *G. kirkii* was three times that in *A. thaliana*. These results showed that the number of *LDAP* genes increased approximately twofold with the formation of *G. kirkii* or *G. raimondii*, and the expansion appears to occur independently in the two species.

### The presumed expansion process that occurred in *G. kirkii* and *G. raimondii*

**Figure 3:**
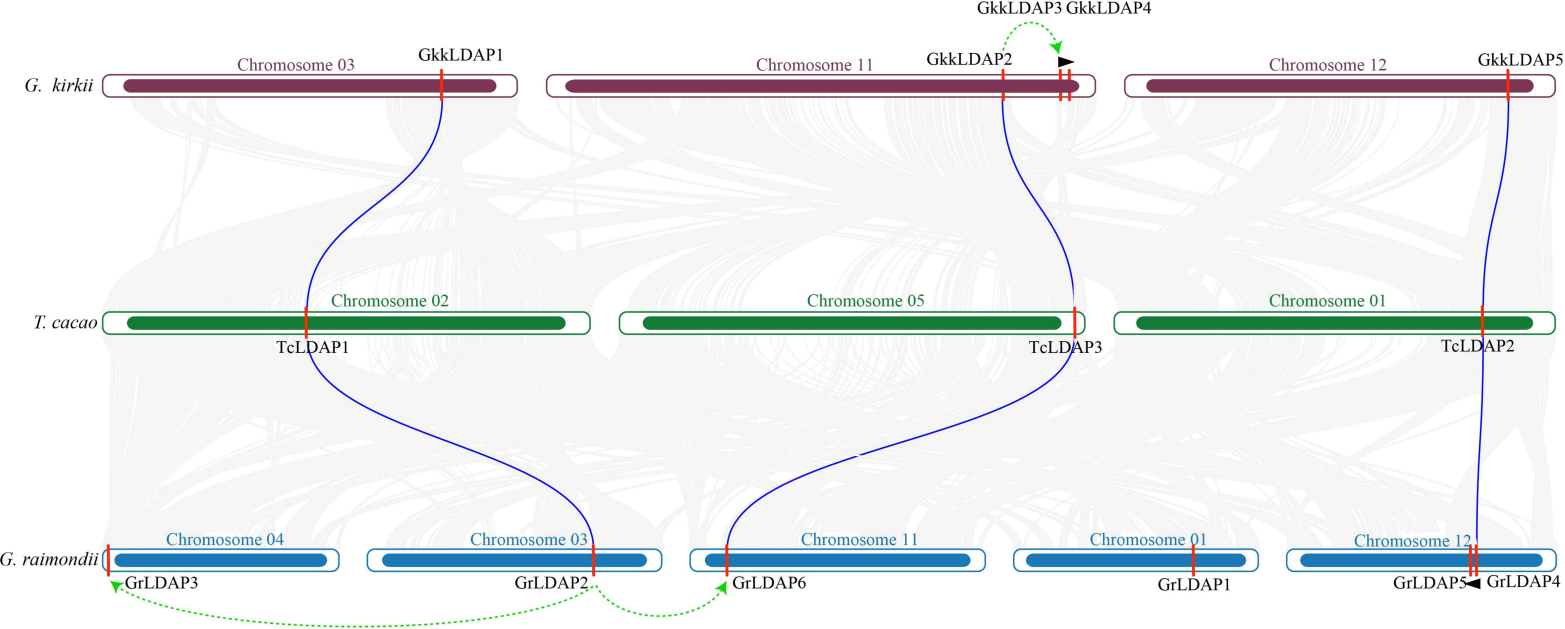
Collinearity analysis and gene duplication events among *G. kirkii*, *T. cacao*, and *G. raimondii*. The two ends of the blue lines are oriented toward the orthologous genes between *G. kirkii* and *T. cacao* and between *G. raimondii* and *T. cacao*, respectively. The green arrows represent segment duplication events that occurred within species, and the black triangles represent tandem duplication events within species.

To further detect the expansion process that occurred in *G. kirkii* and *G. raimondii*, we identified the orthologous genes in these species. Firstly, the LDAP protein sequences in *G. raimondii* and *G. kirkii* were aligned with each other using BLAST, and the result showed that the three *LDAP* genes in *G. kirkii* (*GkkLDAP1*, *GkkLDAP2*, and *GkkLDAP5*) showed the highest sequence similarity (identity >90%) with the three *LDAP* genes in *G. raimondii* (*GrLDAP2*, *GrLDAP6*, and *GrLDAP4*), respectively, while the sequence similarity between the other genes in *G. raimondii* and *G. kirkii* was lower (identity <70%) (**Supplementary Table S5**). In addition, OrthoFinder was used to identify the orthologous genes, and three orthologous gene pairs (*GkkLDAP1-GrLDAP2*, *GkkLDAP2-GrLDAP6*, and *GkkLDAP5-GrLDAP4*) were found between *G. kirkii* and *G. raimondii* (**Supplementary Table S6**). Subsequently, the *LDAP* genes of *G. kirkii* and *G. raimondii* were aligned to those of *T. cacao*, and a collinearity analysis was performed with each other. The result showed that the three orthologous pairs between *G. kirkii* and *G. raimondii* showed the highest sequence similarity to the three *LDAP* genes in *T. cacao* and were located on the larger collinearity blocks between species (**Figure 3**, **Supplementary Table S5**). In summary, the three orthologous pairs in *G. kirkii* and *G. raimondii* are highly conserved during evolution, and they may be derived from the common ancestor of the two species, while other *LDAP* members in *G. kirkii* and *G. raimondii* may have arose from species-specific duplication events. Furthermore, MCSCAN was used to detect the genome duplication events of *G. raimondii* and *G. kirkii* through self-blast. The results showed that *GkkLDAP3* may have arose from ancient member *GkkLDAP2 via* segment duplication, and then a tandem duplication of *GkkLDAP3* occurred to form *GkkLDAP4*. For *G. raimondii*, *GrLDAP3* and *GrLDAP6* may have arose from ancient member *GrLDAP2 via* segment duplication, while *GrLDAP5* may have arose from ancient member *GrLDAP4 via* tandem duplication (**Figure 3**, **Supplementary Table S7**).

### Gene structure and conserved motif analysis of *LDAP* genes

**Figure 4:**
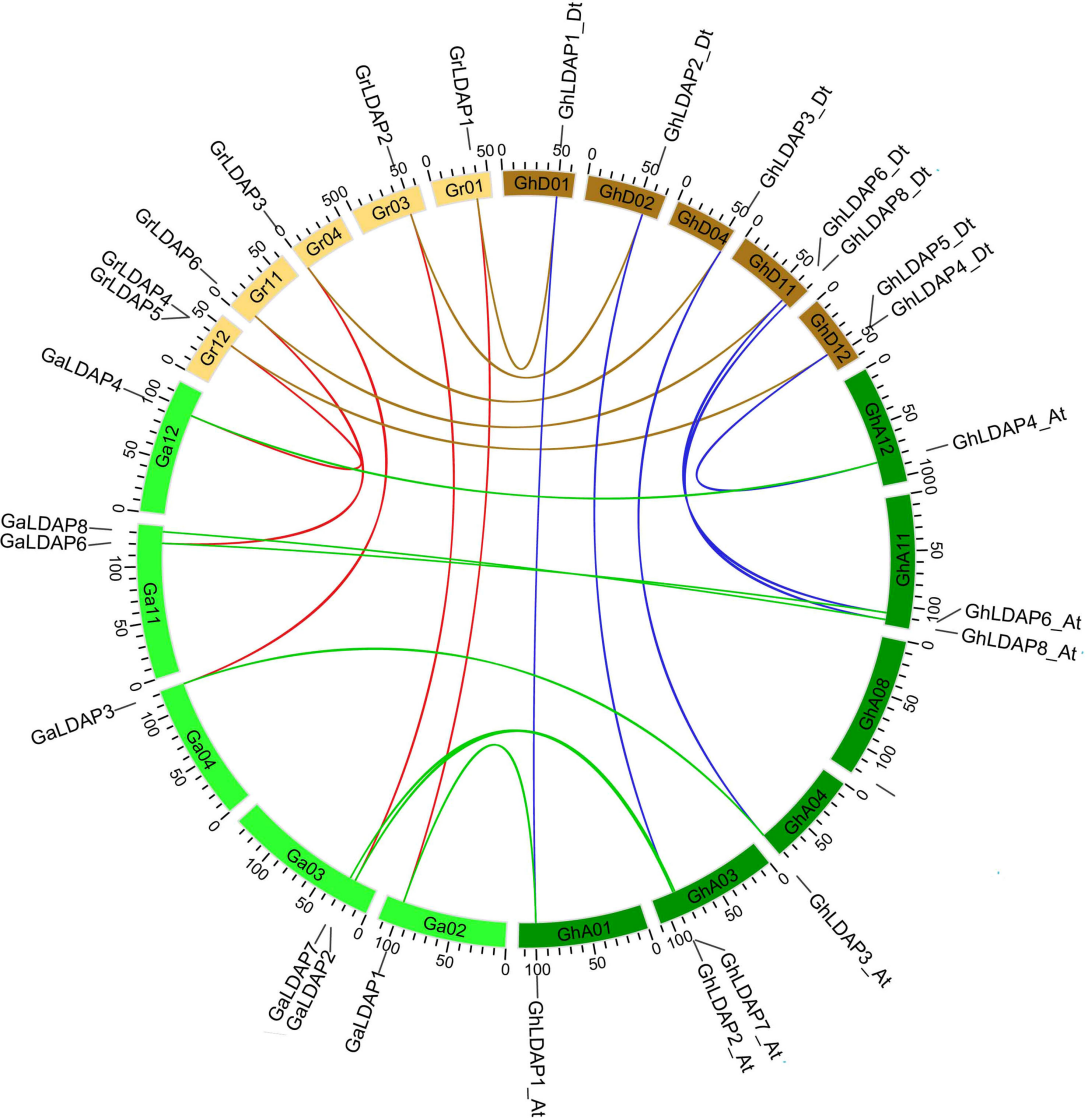
Collinearity analysis of *LDAP* genes among *G. hirsutum*, *G. arboreum*, and *G. raimondii*. The ends of the blue lines are oriented toward the orthologous genes from the At and Dt sub-genomes of *G. hirsutum*. The ends of the green, brown, and red lines are oriented toward the orthologous genes between *G. hirsutum* and *G. arboreum*, *G. hirsutum* and *G. raimondii*, and *G. arboreum* and *G. raimondii*, respectively.

**Figure 5:**
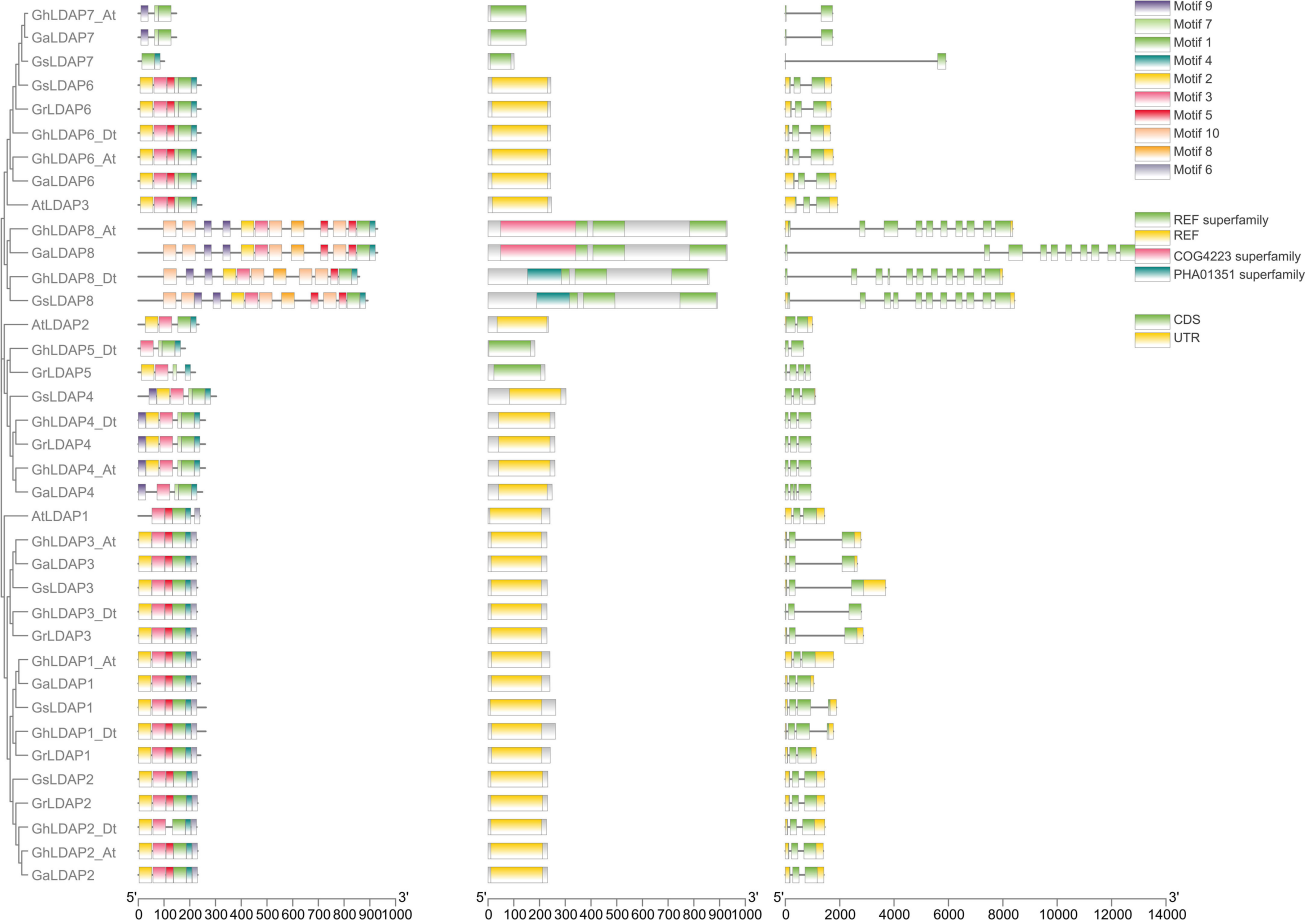
Conserved motif and gene structure of *LDAP* genes in *Arabidopsis* and five cotton species.

To assess the evolution of *LDAP* genes that preceded and followed allopolyploid formation, we performed a collinear analysis of *LDAP* genes among the two diploids (*G. raimondii* and *G. arboreum*) and the tetraploids (*G. hirsutum*) (**Figure 4**). Five, seven, and five orthologous gene pairs were identified between the genomes of *G. arboreum* and *G. raimondii*, *G. arboreum* and *G. hirsutum*, and *G. raimondii* and *G. hirsutum*, respectively, indicating that most of the *LDAP* loci among these species were significantly conserved during cotton evolution. Additionally, their structural diversity analysis was performed based on the Generic Feature Format (gff3) files. As shown in **Figure 5**, *LDAPs* are multiple-exon genes (two to 11 exons), and the genes close to each other in the phylogenetic tree exhibited highly similar exon patterns. The exon pattern for genes in group I were the most similar, where 14 of 16 genes in this group have three exons. However, the exon patterns of genes in groups II and III showed larger variability—for example, the exon numbers in group III ranged from two to 11. Additionally, MEME was used to discover the conserved motifs in *LDAP* genes. In total, 10 motifs were identified in the *LDAP* genes, where motifs 1, 2, 3, 4, 5, and 7 are located in the REF domain (**Figure 5**, **Supplementary Figure S1**). Members of the same subfamily mostly have similar motif components. Motifs 1–4 were present in more than 80% of the genes in group III, motif 5 was mainly present in group I and II members (except for *GaLDAP4*), motif 6 was only present in *LDAP* genes in subgroup I, motif 7 and motif 9 were only present in members of groups II and II, and motif 8 and motif 10 were only present in *LDAP8* genes (**Figure 5**).

### Gene expression patterns of *LDAP* genes

**Figure 6:**
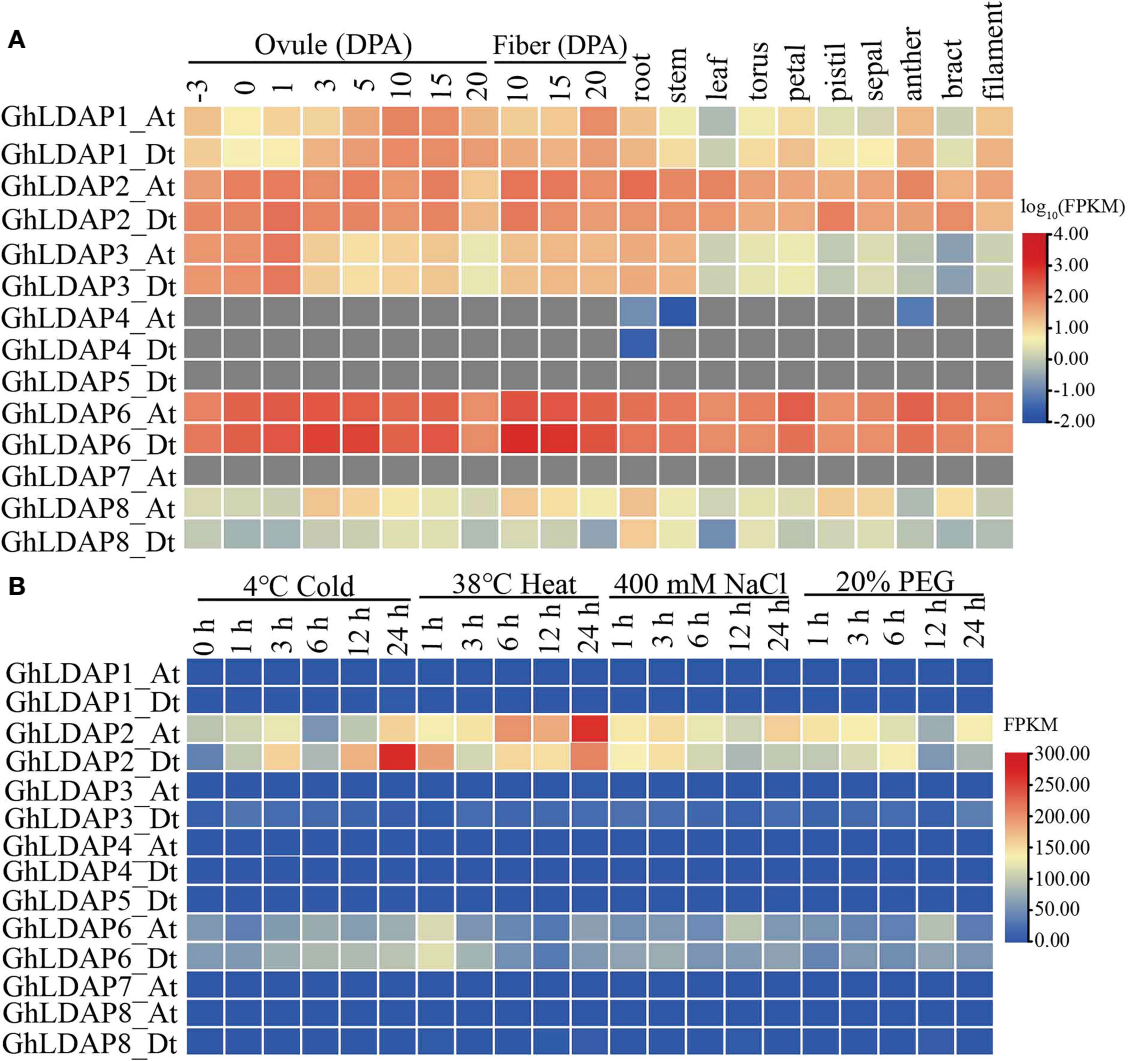
Gene expression profiles of *LDAP* genes in different tissues **(A)** and under different abiotic stresses **(B)**.

To understand the biological function of *LDAP* genes, we examined the *LDAP* gene expression patterns based on the gene expression database of TM-1 (Zhang et al., 2015). As shown in **Figure 6A**, two genes in group I, *GhLDAP2_At/Dt*, showed a high expression level in all reproductive tissues (torus, petal, anther, sepal, bract, and filament) and vegetative tissues (root, stem, and leaves). The other members in group I (*GhLDAP1_At/Dt and GhLDAP3_At/Dt*) were also expressed in various tissues but only at higher levels during ovule development. However, the expression of three *LDAP* genes of group II were not detectable in each tissue (**Figure 6A**). For group III members, *GhLDAP6_At/Dt* showed the highest expression level in all tissues, the expression level of *GhLDAP8_At/Dt* was low in each tissue, while *GhLDAP7_At* was not expressed in various tissues (**Figure 6A**). As fiber is an important economic product of cotton, we also analyzed the expression pattern of *GhLDAPs* in different periods of fiber development. Similarly, the genes with the highest expression levels during the different periods of fiber development were also *LDAP2_At/Dt* and *LDAP6_At/Dt*, among which the FPKM values of *LDAP6_At/Dt* in 10 DPA and 15 DPA fiber were significantly higher than those in other tissues (**Figure 6A**), indicating that they have a potential role in fiber development. Additionally, we also analyzed the expression patterns of *LDAP* genes under different abiotic stresses (PEG, NaCl, cold, and hot) (**Figure 6B**). The results showed that *GhLDAP2_At/Dt* were strongly induced by various abiotic stress environments, and the expression of the *GhLDAP2_Dt* was induced to a greater extent than that of *GhLDAP2_At*.

Based on the RNA-Seq data of *G. stocksii* and *G. arboreum* to PEG-simulated drought stress (Yu et al., 2021), we analyzed the expression patterns of *LDAP* genes in PEG-stressed *G. arboreum* and *G. stocksii*, respectively. The results showed that the expression levels of *GaLDAP2* and *GsLDAP2* were significantly induced in PEG-stressed *G. arboreum* and *G. stocksii*, respectively (**Supplementary Figure S2**). Taken together, the expression levels of *LDAP2* genes in all three cotton varieties (*G. hirsutum*, *G. stocksii*, and *G. arboreum*) were significantly induced after PEG treatment, suggesting a potential role for *LDAP2 in* coping with drought stress. Subsequently, the function of *GhLDAP2_Dt* in response to drought stress was further analyzed.

### *GhLDAP2_Dt* acts as a positive regulator in cotton during drought stress

**Figure 7:**
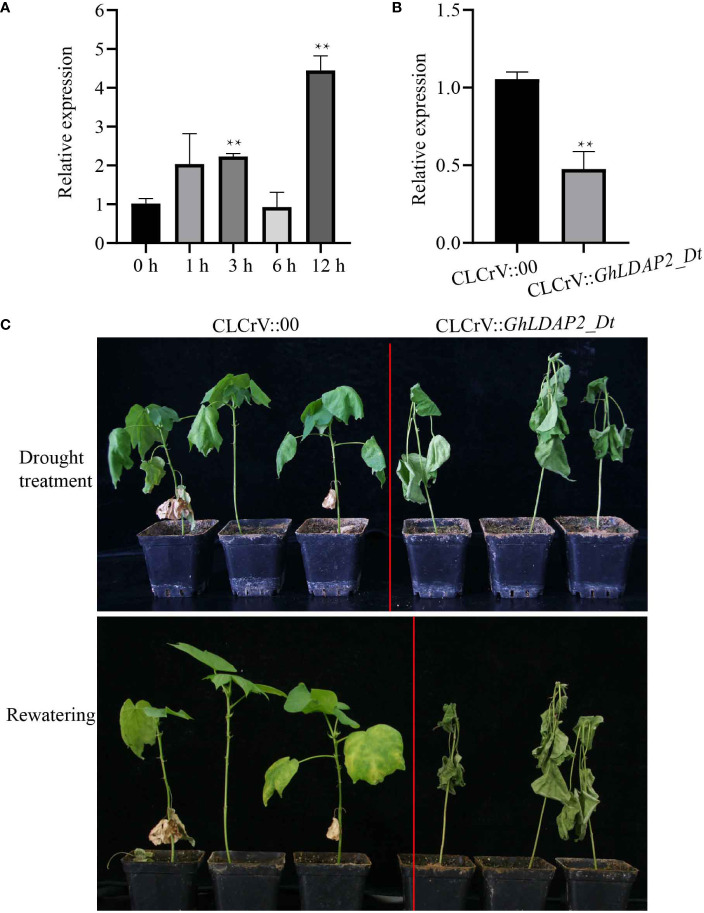
Effect of silencing of *GhLDAP2_Dt* in cotton under drought stress. **(A)** Expression pattern of *GhLDAP2_Dt* in C312 after 20% PEG treatment. **(B)** *GhLDAP2_Dt* expression levels in the leaves of *GhLDAP2*-silenced (CLCrV::*GhLDAP2_Dt*) and control (CLCrV::*00*) cotton. **(C)** Phenotypes of drought-stressed *GhLDAP2*-silenced and control cotton plants. *GhUBQ7* was used as the reference gene. The asterisks indicate statistically significant differences as determined by Student’s two-tailed *t*-test (*P < 0.05, **P < 0.01).

The transcription levels of *GhLDAP2_Dt* following PEG treatment were further confirmed through qRT-PCR in drought-tolerant *G. hirsutum* ‘S08’. The result showed that the transcript levels of *GhLDAP2_Dt* were significantly upregulated at 3 h of PEG-treated plants compared with those of water-treated plants (0 h) and reached a maximum at 12 h of PEG treatment (**Figure 7A**), suggesting that *GhLDAP2_Dt* may play an important role in cotton responses to drought stress. To further confirm the role of *GhLDAP2_Dt* in modulating drought stress in cotton, CLCrV-based VIGS was conducted to interfere with the expression of *GhLDAP2_Dt* and obtain *GhLDAP2_Dt*-silenced cotton. The interference efficiency was assessed using qRT-PCR, and the result showed that the transcript level of *GhLDAP2_Dt* in *GhLDAP2_Dt*-silenced plants (CLCrV::*GhLDAP2_Dt*) was significantly lower compared with that of the control (CLCrV::*00*) (**Figure 7B**). Following the drought treatment for nearly 3 weeks, in the *GhLDAP2_Dt*-silenced plants, we observed chlorosis and wilting, whereas the control plants displayed mild symptoms. After the rewatering treatment, the leaves of control plants fully expanded and returned to normal growth, while the *GhLDAP2_Dt*-silenced plants still remained dry (**Figure 7C**). Thus, reduced transcript levels of *GhLDAP2_Dt* lead to increased sensitivity to drought in cotton.

**Figure 8:**
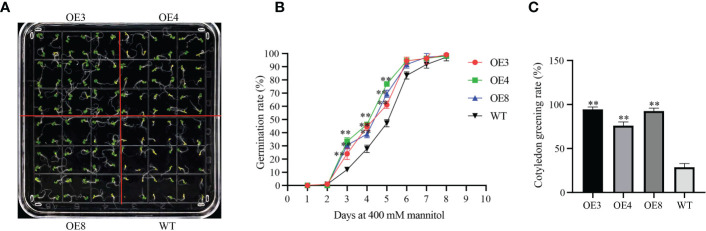
Osmotic tolerance of *GhLDAP2_Dt* transgenic *Arabidopsis*. **(A)** Assay of *GhLDAP2_Dt*-over-expressing (OE) and wild-type (WT) *Arabidopsis* seed germination after 10 days in MS medium supplemented with 400 mM mannitol. **(B)** Seed germination rate on MS agar plates saturated with 400 mM mannitol. **(C)** Cotyledon greening rate for OE lines and WT plants after mannitol treatment. Asterisks indicate statistically significant differences, as determined by Student’s two-tailed t-test (*P < 0.05, **P < 0.01).

In addition, we overexpressed *GhLDAP2_Dt* in *Arabidopsis*. Three overexpression lines (OE3, 4, and 8) with high transcript levels of *GhLDAP2_Dt* were used for further studies. Moderate or severe drought also imposes osmotic stress, and the tolerance of OE lines to mannitol-simulated osmotic stress was determined during germination stage, and tolerance analysis was performed by calculating the seed germination rate and cotyledon greening rate. When germinated on normal MS medium, the plant phenotype showed no significant difference among WT and OE lines. However, when these lines were germinated on MS medium containing 400 mM mannitol, the WT displayed delayed germination initiation and grew more slowly than the OE lines did (**Figures 8A, B**). When treated with 400 mM mannitol for 10 days, the cotyledon greening rate of OE seedlings was significantly higher than those of WT seedlings (**Figures 8A, C**). Thus, in transgenic *Arabidopsis* with overexpressed *GhLDAP2_Dt*, it was observed that there was enhanced resistance to mannitol-simulated osmotic stress.

## Discussion

Recent studies have shown that LDAP proteins play an essential role in plant growth, development, and response to abiotic stresses (Horn et al., 2013; Gidda et al., 2016; Pyc et al., 2017). With the development of genome sequencing technology, whole-genome analyses of the *LDAP* gene family have been performed in several plants, including *Hevea brasiliensis*, *Taraxacum brevicorniculatum*, and *A. thaliana* (Kim et al., 2016; Tang et al., 2016; Laibach et al., 2018). Cotton is an important fiber and cash crop (Sun et al., 2022); however, the LDAP family has not been characterized in cotton. Nowadays, high-quality whole genomes of *G. hirsutum*, *G. arboreum*, *G. raimondii*, and *G. stocksii*, respectively, have been better sequenced (Yang et al., 2019; Wang et al., 2021; Yu et al., 2021). With this, we identified and characterized the *LDAP* genes in cotton to gain insight into their roles therein.

Allopolyploid cotton may have appeared through hybridization and subsequent polyploidization events between the A- and D-subgenome progenitors that occurred in the last 1–2 million years (Li et al., 2014; Huang et al., 2020). Upland cotton is a typical allotetraploid and offers a powerful model to study polyploidy formation in evolution. In this study, a total of 13, six, and seven *LDAPs* were identified in *G. hirsutum*, *G. raimondii*, and *G. arboreum* (**Figure 1**). The number of *LDAPs* in tetraploid cotton *G. hirsutum* is close to the total number in diploid cotton *G. raimondii* and *G. arboreum*, and the *LDAPs* of tetraploid corresponded to *LDAPs* of diploid one by one and were clustered together within each sub-group in the phylogenetic tree (**Figures 1**, **4**). These results indicated that the LDAP gene families of the A- and D-genome in diploids combined and formed the LDAP gene family in neoallopolyploids, which occurred during the hybridization and polyploidization of two diploid progenitors to form the allopolyploid cotton.

We also identified the *LDAP* gene family members in species other than the *Gossypium* genus to elucidate the evolutionary history of the LDAP gene families. Gossypioide species *G. kirkii* is a sister species to *Gossypium* (Wang et al., 2020), and similar LDAP gene numbers were identified among *G. kirkii* and two diploid *Gossypium* species (not less than five), while other species, such as *T. cacao*, *C. capsularis*, *C. papaya*, and *A. thaliana*, only have three LDAP genes (**Figure 2A**), indicating that the *LDAP* family expansion occurred accompanied with the formation of *Gossypium* species. The earliest plants had one or only a few ancient genes, and the genes of modern plants descended and radiated from these predecessors by gene duplications (Carretero-Paulet et al., 2010; Feller et al., 2011). Combining the results of BLASTP, OrthoFinder, and MCSCAN, we identified three ancient members in *G. raimondii and G. kirkii*, respectively, and other members in *G. raimondii and G. kirkii* were generated after the divergence between *G. raimondii and G. kirkii* through species-specific duplication events (**Figure 3**). *LDAPs* share a high sequence similarity with the *SRPPs* found in rubber-accumulating plants (Horn et al., 2013). Previous studies have suggested that the expansion of the REF/SRPP family in rubber-producing plants may correlate with their rubber production capacity (Tang et al., 2016). The expansion of the LDAP family in *Gossypium* indicated that the LDAP family members act an essential role in the development of cotton; however, what exactly that role is needs to be further explored.

It has long been thought that gene duplication can lead to at least three functional outcomes, including neofunctionalization, subfunctionalization, and non-functionalization (Feller et al., 2011; Zhang et al., 2020). The analysis of gene structure and conserved domains showed that *LDAPs* derived from group I (*GhLDAP1*/*2*/*3*) are highly conserved during evolution (**Figure 5**), and the gene expression pattern of these genes showed that they are expressed in all tissues, especially in ovules where they were highly expressed (**Figure 6**), indicating that these genes are functionally conserved during evolution. Members from group II, *GhLDAP4* and *GhLDAP5*, had large variations in both domain and gene structure, and their gene expression pattern showed that they are not expressed in all tissues (**Figures 5**, **6**), indicating that they are not conserved during evolution and have undergone non-functionalization. Similarly, members from group III, *GhLDAP6*, *GhLDAP7*, and *GhLDAP8*, also had large variations in both domain and gene structure, and the gene expression pattern analysis showed that, except for *GhLDAP6*, which is highly expressed in all tissues, all other genes were barely expressed in all tissues, indicating that these genes undergo large mutations resulting in non-functionalization during the duplication process.

Because LDs are mainly present in seeds, studies on LD-associated proteins, especially oleosins, in higher plants have mainly focused on seed development and germination (Lévesque-Lemay et al., 2016; Shao et al., 2019). The plant seed is an organ formed by the maturation of the ovule in flowering plants. In this study, the gene expression pattern analysis of *LDAP* genes showed that they are expressed not only in reproductive organs (ovule) but also in vegetative organs (root, stem, and leaves) (**Figure 6**). The broader gene expression pattern of *LDAPs* suggested a more general function in the entire plant life cycle for *LDAPs* rather than the major role for *oleosin*s in maintaining stability during seed development. Fiber yield and quality are the most important production traits in cotton, and mining functional genes that can regulate cotton fiber development is an important way to improve cotton fiber quality (Ke et al., 2022). In this study, we found that the expression level of *GhLDAP6_At*/*Dt* was significantly higher in fiber development than in other tissues, indicating that it may be an important regulator of cotton fiber development. The function of LDAP family genes during cotton fiber development has not been reported. The present study provides a new excellent candidate gene for cotton fiber improvement, and its function in cotton fiber development needs to be further verified by obtaining its stable genetic material subsequently. In other species, *LDAPs* have also been reported to be involved in abiotic stress in plants (Kim et al., 2010; Kim et al., 2016)—for instance, *AtLDAP1*–*3* overexpression transgenic plants exhibited better drought tolerance than wild-type *Arabidopsis* (Kim et al., 2016). Considering the close involvement of *LDAPs* in abiotic stress, we identified the *LDAPs* induced by the abiotic stress in *G. hirsutum* and found that *GhLDAP2_At/Dt*, especially *GhLDAP2_Dt*, were strongly induced by various abiotic stress environments. Decreasing the expression of *GhLDAP2_Dt* in cotton *via* VIGS increased the drought sensitivity and over-expression of *GhLDAP2_Dt*, leading to increased tolerance to mannitol-simulated osmotic stress at the germination stage. Thus, we conclude that *GhLDAP2_Dt* plays a positive role in drought tolerance, which is potentially useful for engineering drought-tolerant cotton.

## Data availability statement

The original contributions presented in the study are included in the article/**Supplementary Materials**. Further inquiries can be directed to the corresponding author.

## Author contributions

YZ and YS conceived the research plans. Material preparation, data collection, and analysis were performed by BD, YL, DY, and YW. NZ conducted DNA and RNA extractions and PCR analysis. YZ and JM wrote the first draft of the manuscript. YZ, FC, and LK revised the manuscript, and all authors commented on previous versions. All authors contributed to the article and approved the submitted version.
