# Supplementary figures and images for: Identification and characterization of the LDAP family revealed GhLDAP2_Dt enhances drought tolerance in cotton
Source: Front Plant Sci. 2023 May 16;14:1167761. doi: 10.3389/fpls.2023.1167761 (PMC10228748; doi:10.3389/fpls.2023.1167761)

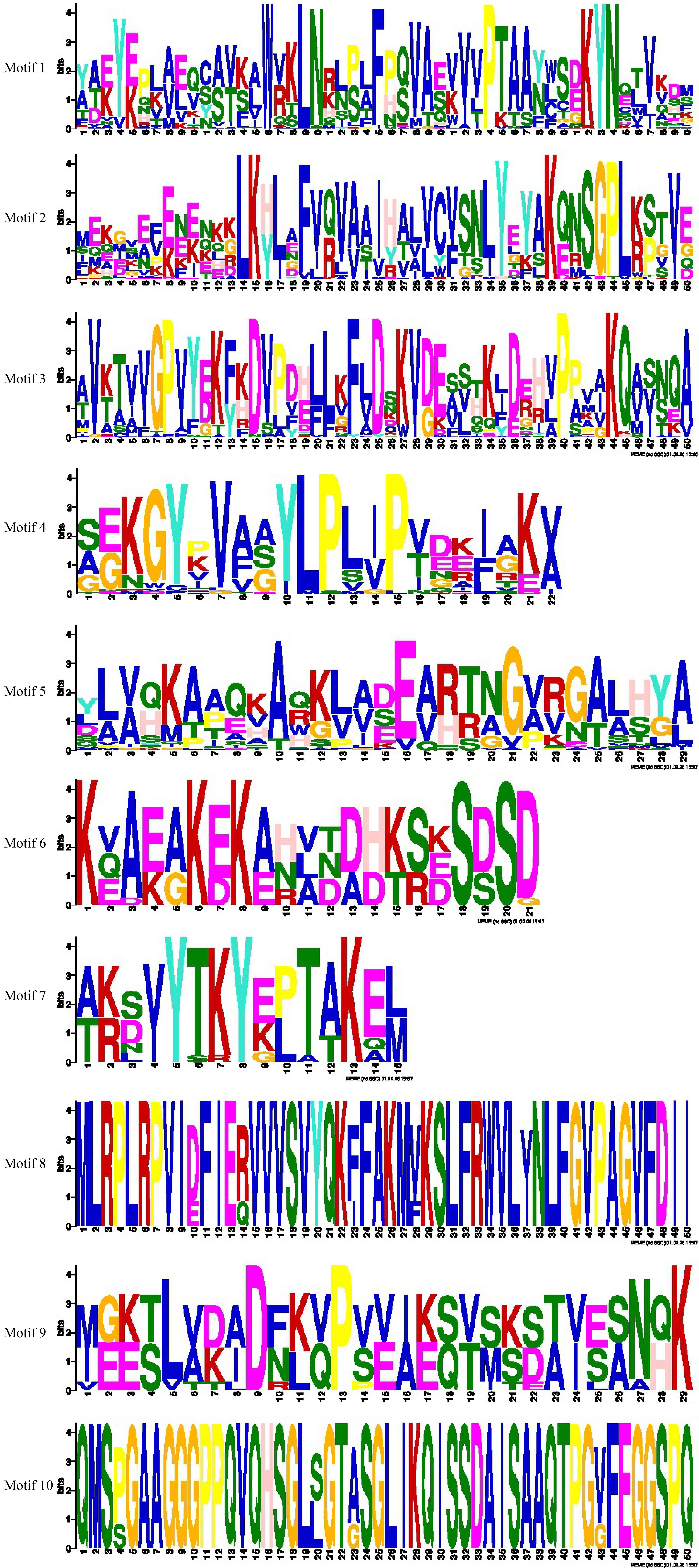

Supplement: Supplementary file 1 [file Image_1.jpeg]

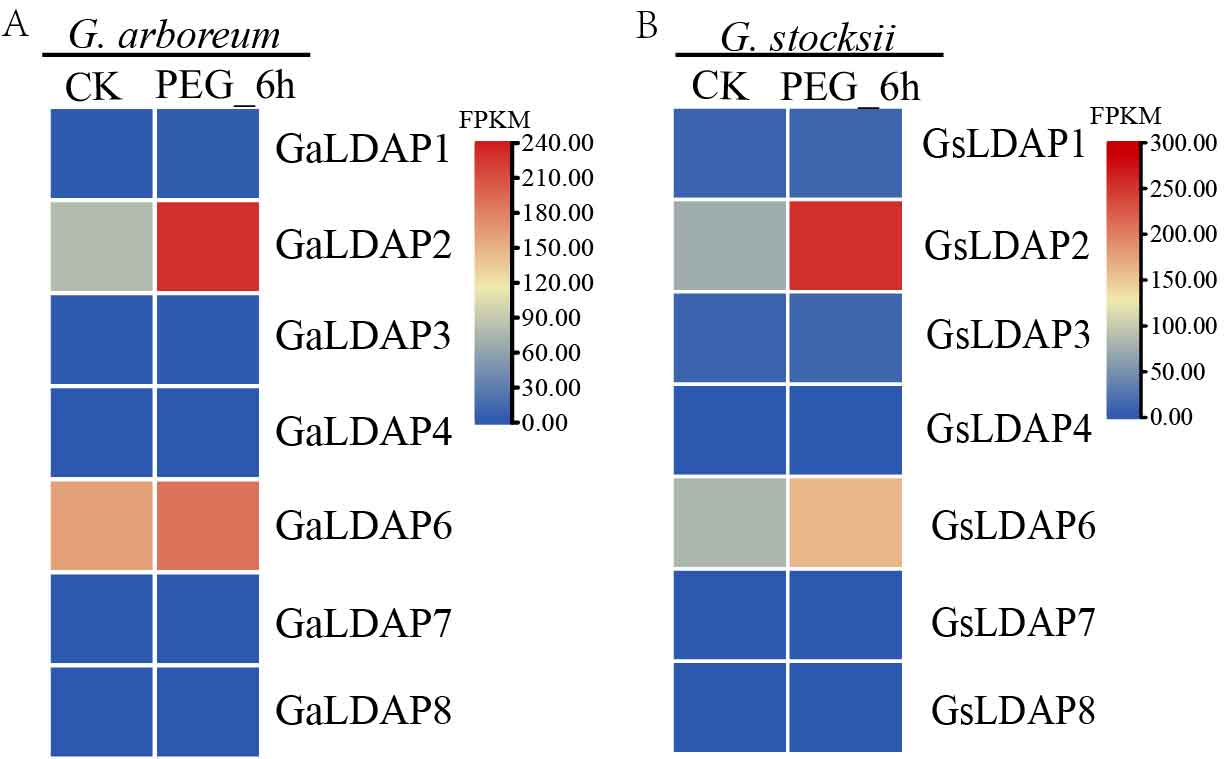

Supplement: Supplementary file 2 [file Image_2.jpeg]
